# Residential proximity to oil and gas development and socioeconomic conditions: A comparative analysis of 33 countries

**DOI:** 10.1088/1748-9326/aeae53

**Published:** 2026-10-23

**Authors:** Lara Schwarz, Nina M. Flores, David J.X. González, Heather McBrien, Nina Brooks, Jonathan J. Buonocore, Amira Aker, Veronica Southerland, Joan A. Casey, Mary D Willis

**Affiliations:** 1 Division of Environmental Health Sciences, University of California Berkeley School of Public Health, Berkeley, CA, USA; 2 Department of Environmental Health Sciences, Mailman School of Public Health, Columbia University, New York, NY, USA; 3 School for Environment and Sustainability, University of Michigan, MI, USA; 4 Department of Environmental Health, Boston University School of Public Health, Boston, MA, USA; 5 Department of Epidemiology, Boston University School of Public Health, Boston, MA, USA; 6 Environmental Defense Fund, USA; 7 Department of Environmental Health and Occupational Health Sciences, School of Public Health, University of Washington, Seattle, WA, USA; 8 Department of Epidemiology, School of Public Health, University of Washington, Seattle, WA, USA

**Keywords:** fossil fuels, extraction, population, health

## Abstract

Fossil fuels continue to dominate the global energy supply. As emerging technologies drive up global energy consumption (e.g., cryptocurrency mining, data centers), identifying which populations live in residential proximity to oil and gas extraction will become increasingly important. Yet, no prior study has done so on an international scale. We combine the Oil and Gas Infrastructure Mapping database with the Global Human Settlement Layer to estimate residential proximity to oil and gas wells across 33 countries in 2024. We further link these data with indicators of income and social deprivation to examine disparities in population proximity. We estimate that 150.9 million people reside within 3 km of 4.5 million oil and gas wells across 33 countries, representing 10% of their population. Country-level residential proximity to oil and gas wells is highest in the Netherlands (38.4%), Canada (33.4%), and the United States (31.3%). Of the 33 countries, 15 had populations exposed to wells across borders, including 427 thousand Mexican residents near U.S. wells, and 185 thousand U.S. residents living near wells outside national borders. This is the first large-scale, multi-country quantification of residential exposure to oil and gas extraction, providing an evidence base for future population health research and policies to reduce risks and address energy-related inequities worldwide.

## Introduction

Fossil fuels are the leading source of energy worldwide, comprising nearly 85% of global primary energy (1). By the end of the century, global energy demand is expected to triple (2). Rising demand will be driven by energy-intensive digital innovations (e.g., cryptocurrency mining, artificial intelligence, hyperscale data centers) (3–5). Meanwhile, the geography of global energy demand is rapidly shifting. In 2007, total energy consumption was roughly equivalent among member and non-member countries of Organisation for Economic Co-operation and Development (OECD, members of which are predominantly high-income nations); however, by 2035, energy use in non-OECD countries is projected to rise by 84%, compared to a 14% increase in OECD countries (6). While some transition towards renewable energy will occur, oil and gas is expected to remain the dominant global energy source through 2035 (7).

While the oil and gas industry continues to dominate global energy production, living in proximity to oil and gas wells is consistently associated with adverse population health outcomes (8–13). Oil and gas extraction creates air pollution and water contamination at levels that harm human health and the local environment (14). These exposures are associated with a range of adverse health outcomes (e.g., birth defects, pregnancy complications, asthma exacerbations, mortality) (10,15). In the United States (U.S.) alone, across the life cycle of oil and gas, this industry has annual burdens of 91,000 premature deaths, 10,350 preterm births, 216,000 incidences of childhood-onset asthma, and 1,610 lifetime cancers from hazardous air pollutants (16). However, outside of the U.S., little is known about exposure to oil and natural gas and its population health impacts. Estimates suggest that 0.2 million premature deaths annually are attributable to fine particulate matter air pollution from fossil fuels (17).

Economics and trade literature demonstrate that oil and gas production affects populations on a global scale. Across over 40 countries on six continents, there are 1.57 trillion barrels of crude oil reservoirs as of 2023, and a 2.6% increase in global oil demand since 2022 (18). Over the next 15 years, the Middle East and North America are projected to contribute half of the global supply (7). In contrast, Europe’s share of the global oilfield market is expected to decline and the shares from Asia, Africa, and Latin America will remain relatively stable (7). These trends indicate that the harms of oil and gas may increasingly burden residents of low- and middle-income countries, further entrenching global inequities and externalizing environmental health burdens.

A growing body of evidence documents the environmental injustices surrounding oil and gas extraction, with most research to date confined to high-income countries (19–21). Oil and gas extraction often occurs close to residential communities, and persistently marginalized groups (e.g., racial and ethnic minority, lower income) are often disproportionately exposed to the industry (22). Coined “fossil fuel racism”, this pattern has been consistently documented in the U.S. and Canada, with emerging evidence of similar patterns in the Amazon region of Brazil, Peru, and Ecuador (14,19,23–27). Since persistently marginalized populations are often already burdened with social stressors, the environmental hazards from oil and gas development can amplify adverse health outcomes in these communities (28). However, nearly all studies on community impacts of oil and gas are from the U.S. and Canada (10), omitting the vast majority of countries with substantial oil and gas industries.

Environmental governance and community participation in political decision-making at local, regional and national levels may be important in influencing the unequal distribution of environmental burdens and benefits, as well as determining what communities experience the greatest harm. Political leadership and regulation can influence the siting and permitting of oil and gas extraction sites and, consequently, the distributive justice of this industry. Although some regions may experience economic benefits from oil and gas development, affected communities are often excluded from planning and decision-making processes, receiving few benefits while disproportionately bearing the environmental and health risks associated with extraction (29).

To understand what communities may be at risk of adverse health outcomes associated with oil and gas wells, we first need to document the geographic scope of where this industry is located and who lives in close proximity. A recently developed open-access multi-country dataset of oil and gas wells, the Oil and Gas Infrastructure Mapping (OGIM) enables analysis of residential proximity to oil and gas wells and social disparities from an international perspective (30). While any multi-country analysis of oil and gas wells underestimates the total exposure, OGIM provides a platform of high-quality data to quantify the scope of the industry from a population perspective.

Here, we leverage this novel dataset to quantify the number of people living near oil and gas wells across 33 countries and assess social disparities. Specifically, we: 1) estimate the number of people living in close proximity to oil and gas wells across these 33 countries; 2) identify residential proximity to oil and gas wells located in neighboring countries; and 3) assess how income and social deprivation shape the distribution of residential proximity to oil and gas wells within and across countries. By integrating multiple data sources across diverse geographic and socioeconomic contexts, this work advances understanding of international patterns and social inequalities in residential proximity to oil and gas extraction.

## Methods

### Data sources

We integrate several data sources into a novel, cohesive entity for this study. Below, we summarize the important qualities of each data source. Further details are described in Table S1.

We use the Oil and Gas Infrastructure Mapping (OGIM) Database v2.5.1 to identify oil and gas wells, which approximates the 2024 infrastructure. This dataset is a curated collection of publicly available geospatial sources of oil and gas facilities (30). Here, we use the data from the ‘oil and natural gas wells’ layer to estimate residential proximity to oil and gas wells. Several countries (n=64) that produce oil and gas are not included in this data set, thus omitted from our analysis (Figure 1) (31). In addition to the 26 countries with oil and gas wells identified in the OGIM dataset, we included countries defined by our geographical data (Papua New Guinea and South Sudan), as well as any country with oil and gas wells located near its border or with populations living in proximity (within 5 km) to an oil and gas well, even when the well was located in a neighboring country, resulting in a total of 33 countries. We estimate that the included countries comprise a little over 50% of total global production. Importantly, the top five countries with the highest number of wells (U.S., Canada, Argentina, Mexico, Australia) have 97% of the total global oil and gas wells in the data set (Table S2). The top five oil producing countries which are not included in the OGIM dataset are Russia, China, Iraq, Iran and Kuwait. A comparison of the OGIM dataset to other available data on oil and gas wells in the U.S. found it to be of high quality (32).

We use gridded population data from the 2020 Global Human Settlement Layer (GHSL), a five-year estimate of residential population produced by the European Commission’s Joint Research Centre to estimate residential proximity to oil and gas wells (33). The GHSL is created using raw census and harmonized with the Gridded Population of the World, version 4.11 to estimate population distribution at a spatial resolution of 100 m. This is a globally consistent, high-resolution raster dataset estimating the distribution of the resident population across the world.

We use data from the Institute for Health Metrics and Evaluation to capture local and small area estimation of lag-distributed income (LDI) at the subregional level globally for 2019 (34). These estimates use a small area estimation modeling process capturing wealth variables such as household income, expenditure, and consumption, asset ownership, and gross domestic product from major survey series. Integrated survey data, predictive covariate, and geospatial modeling are used to predict LDI, accounting for spatial dependence. LDI is a measure of income defined as a moving average transformation of per person income with a 10-year lag globally and is expressed in 2010 purchasing power parity-adjusted dollars provided at the second-level administrative division, such as county or district (ADM2) (34).

We use the Global Relative Deprivation Index (GRDI), at about ~1 km spatial resolution, to estimate global relative levels of poverty. GRDI estimates are based on sociodemographic data and satellite data ranging from 2010 to 2020, with a value of 100 representing the highest level of deprivation and a value of 0 representing the lowest social deprivation (35). This index is built from six input components including measures of rurality, child dependency ratios, infant mortality rates, subnational human development index including education health and standard of living, nighttime lights and changes in nighttime light through time. This gridded dataset is used to estimate average deprivation of populations living near oil and gas wells. We used World Bank Group Income classifications to group countries by income level. We classify countries as “high income” or “low and middle income” (36). To evaluate the potential role of regulatory governance, World Bank Global Indicators of Regulatory Governance, an indicator of transparency, civic participation and government accountability is used for each country (37).

### Statistical analysis

We consider any point labelled ‘oil and natural gas wells’ in the OGIM dataset as an oil and gas well. This classification includes wells regardless of status, so our oil and gas wells dataset included active, idle, and abandoned wells. Distance buffers around extractive sites were used as a proxy for potential exposure to pollutants and other environmental contaminants. Residential proximity has been widely used in the epidemiological literature to characterize the health impacts of oil and gas extraction, with adverse health effects observed among populations living within several kilometers of oil and gas development (38–41). Considering the range of distances used across previous studies, we determined that 3 km represented a relevant middle range for characterizing residential proximity to extractive sites. We additionally considered populations living in closer proximity (1 km) and somewhat farther away (5 km), as different distances may capture different levels and pathways of exposure, including air pollution, noise, and broader social and environmental impacts. We generate distance buffers around each oil or gas site and overlaid the buffers with our global shapefile to identify any country with population living within each buffer of an oil and gas well. The analytic dataset for our study included 33 countries.

We also identify off-shore wells by intersecting each buffered country shapefile and a land/water mask derived from MODIS (MOD44W V6). We consider oil and gas wells in inland water bodies and coastal water bodies as being off-shore (42). Oil and gas wells that are within the buffer distances but outside of the country boundaries and onshore are considered out-of-country wells that are in residential proximity to populations across binational borders.

We use the python package *popexposure* to estimate the total number of people living within 1 km, 3 km, and 5 km of oil and gas wells in each country by county or district, or ADM2 unit (43). We estimate GRDI within each buffer around oil and gas wells and averaged these estimates within each ADM2 unit. To understand the association between income and residential proximity to oil and gas wells, country-level income and residential proximity are compared between countries within each World Bank Income Group. Within-country analyses are conducted by evaluating the distribution of GRDI across quartiles of residential proximity to oil and gas in ADM2 units for each country. To evaluate the potential role of regulatory governance, the World Bank Global Indicators of Regulatory Governance was split into countries into higher regulatory governance (global indicator of regulatory governance ≥3.5) and lower regulatory governance (global indicator of regulatory governance <3.5). Analyses are conducted using Python version 3.11.13. The code is provided at the following link: https://github.com/nina-flores/ogd.

## Results

Across the 33 countries with oil and gas wells, we count 4.5 million oil and gas wells within 5 km of shore in 2024 (Figure 1). There is wide variation in the proportion of population living in close residential proximity to oil and gas wells by country, with some countries showing over a quarter of their population living within 3km of a well. For instance, the countries with the highest percentages of population in residential proximity to wells were the Netherlands (39.6%), Canada (29.5%), and the U.S. (27.4%) (Table 1). In contrast, out of those included, the countries with the lowest percentage of close residential proximity to wells are Guatemala, Norway, and Sudan (all with <0.01%). At the subnational county and regional level, 4,294 ADM2-level units are in close residential proximity to oil and gas wells, of which 2,744 are in high income countries and 1,550 in low- and middle-income countries (Figure S1). Of the 33 studied countries (Figure 1), 16 have offshore oil and gas wells within 3 km of the coastline (Table S3). We find that 150.9 million people live within 3 km of an oil and gas well across all study countries, a distance that is often associated with increased health risks per epidemiologic literature.

A total of 15 countries had populations exposed to wells in neighboring countries (i.e., these populations are close to oil and gas wells outside of their own country’s boundary). The highest out-of-country residential proximity to wells is in Mexico, where 427,033 residents, or 0.3% of the total country population, were exposed to a well located in the U.S. The U.S. has the second highest out-of-country well residential proximity with 78,381 people living within 3km of a well located in Mexico and 106,217 residents exposed to a well in Canada. Notably, the entire population in France living in proximity to an oil and gas well (10,532) is from wells located in Germany as the OGIM dataset includes no oil and gas wells within France (Figure 2, Table S3).

We observe differences in the association between income or social deprivation and the distribution of residential proximity to oil and gas wells within and across countries. When countries were grouped by World Bank income classification, greater national income corresponded with lower residential proximity to wells in high income countries, while greater income corresponded with higher residential proximity to wells in low- and middle-income countries (Figure 3). The broad trend remains similar across different proximity criteria (1 km, 3 km, 5 km), though different countries emerge as the most highly exposed depending on the buffer distances (Figure S2).

When examining within-country residential proximity to oil and gas wells with respect to income, there is variation in the observed trend across countries (Figure 4). In certain high-income countries (e.g., Australia, Denmark), we observe a decreasing trend in income as oil and gas well residential proximity increased within sub-national units (i.e., ADM2), while other high-income countries show no change (e.g., the U.S.) or no specific pattern (e.g. Italy, United Arab Emirates) emerges. In some low- and middle- income countries (e.g., Argentina), we see income increasing as the population within closer residential proximity of oil and gas increases. However, no consistent pattern is observed in other countries (e.g., Brazil, Colombia, Venezuela). Trends are broadly similar for the 1 km and 5 km buffer (Figure S3 and S4). When comparing across broad country income groups, no specific pattern emerges (Figures S5).

The association between average gridded relative deprivation index (GRDI) and residential proximity to oil and gas wells for different distances from wells substantially varies by country. Generally, we find a flattening slope as the distance increases for both high income and low- and middle- income countries (Figure 5). Results by country are similar; for example, Venezuela shows an increasing slope for the best fit line for both 1 km and 3 km distance, indicating a positive association between residential proximity to oil and gas wells and deprivation, while the 5 km buffer shows a negative association (Figure S6). Broad patterns by country income status or other geographic characteristics are not easily discernible. When grouping countries by lower and higher regulatory governance, those with lower regulatory governance show slightly increasing association between income and percentage population living within 3km of an oil and gas well, while those with higher regulatory governance do not show a relationship with income (Figure S7).

## Discussion

This study represents an assessment of residential proximity to oil and gas wells and related social disparities across 33 countries. Our results reveal associations between residential proximity to oil and gas wells, income, and social deprivation that vary across countries and regions. We estimate that 150.9 million people, or 10% of the population in these 33 countries, live within 3km of an oil and gas well, with 59.6 million, 4%, within 1km of a well. Importantly, epidemiologic literature consistently finds increased risks of adverse health outcomes within 3km of oil and gas wells, warranting future evaluations of these populations (10). Among these residents, 7.6 million live near an offshore well and 0.9 million live near a well located in a neighboring country. These numbers represent a substantial global health concern, particularly as energy demand continues to rise and environmental health disparities persist and deepen worldwide (44), highlighting the need for a broad research and policy agenda on the health and equity implications of global oil and gas development.

High-income countries benefit from energy consumption while externalizing extraction-related harms to lower-income regions, a dynamic driven by neocolonialism that has been central to the global oil and gas trade (45). Yet, evidence on health effects of proximity to oil and gas wells remains concentrated in the few countries, primarily the U.S. and Canada, that have developed a sustained research agenda around this topic (10). Our estimates of residential proximity to oil and gas wells add evidence to this existing research. A U.S. study found that 20 million people live within 1.6 km of an active oil and gas well (46). We find that 42.8 million U.S. residents live within 1 km of any oil and gas well, and 91.9 million within 3 km. Our estimates are substantially larger than the prior estimate, driven in part by our inclusion of all oil and gas wells, regardless of status.

Existing research estimated that oil fields impact the health and environment of over 600 million people in low- and middle-income countries (47). However, this work relied on global oil reservoirs data for their estimates, an imprecise proxy for extraction locations. We build on this work with more detailed and regionally specific quantification of populations residing near extractive sites in an expanded region across countries from various income regions (9,14). Our work fills gaps identified in the literature. For example, a recent review described the dearth of evidence from countries that produce high volumes of oil or gas, such as Saudi Arabia, Mexico, and the United Arab Emirates (14). We estimate residential proximity to oil and gas wells, identifying 40,378 exposed residents in Saudi Arabia, 8 million in Mexico, and 90,946 in the United Arab Emirates (UAE). However, these results represent a vast underestimation, as Saudi Arabia and the UAE are some of the highest oil-producing countries. Our findings, in combination with this previous work, underscore the potential health burden of proximity to oil and gas development for a large population beyond high-income countries. Consistent, high-quality data on the oil and gas well locations, characteristics and population exposure are needed on a global scale.

Furthermore, we find that almost one million people live near oil and gas wells outside of their own country, underscoring the transboundary nature of this industry. The largest cross-national residential proximity was observed along the U.S.-Mexico border, where wells located in the U.S. are within 3 km of an estimated 427,033 residents in Mexico. On the other side of the border, wells located in Mexico are within 3 km of 78,381 U.S. residents. This highlights the importance of cross-border collaboration in managing these sites and their potential health and equity concerns.

We observe that the relation between oil and gas residential proximity and socioeconomic status varies across and within countries, with little clear patterning by shared characteristics. For example, data from Australia and Denmark show that closer residential proximity to oil and gas wells appears to be linked to lower income, whereas the data from Argentina, Bolivia, Canada, Mexico and the United Kingdom show that higher residential proximity to wells appears to be associated with higher income. Using the gridded deprivation index, we also find that associations between residential proximity to wells and deprivation weakened with increasing distance from wells. Distributive injustices around oil and gas development are context specific and shaped by distinct social, political, and historical factors. For instance, previous work in several U.S. states identified exposure disparities related to populations living near oil and gas development, with disproportionately high residential proximity to wells associated with higher poverty, an inequitable distribution of risks and benefits from development (25,48–50). Similar work from Peru identified that poor areas often bear the negative impact of development without seeing benefits (51). The negative association we observed between income and population living in proximity to oil and gas in some of the studied countries and not others demonstrate the need for policies that are tailored to the specific country’s cultural and social context.

There are also potential economic benefits of this industry, such as job creation, income generation, and increased average wages (52). Our results from Mexico show that regions with greater residential proximity to oil and gas wells tended to have higher income, aligning with prior research in Mexico’s Tampico-Misantla Basin that found communities closer to wells had lower marginality (53). Previous evidence has shown that Queensland, Australia experienced net economic gain from gas development, as there are careful regulations regarding the employment of residents, direct social investment, and the distribution of infrastructure grants received by local governments and funded by resource royalties (52). Yet, oil and gas development has been shown to exacerbate procedural inequities; studies in the U.S. have found that most royalty payments often go to nonlocal recipients, leaving nearby residents, who bear the majority of environmental and health burdens, without economic benefits (54,55). Additionally, the distribution of opportunities varies according to proximity to town, town size, capability, and previous exposure to the industry (52). The variation in our results underscore the need to consider how socio-political factors shape fossil fuel-related justice issues across oil- and gas-producing regions worldwide. Our analysis of county-level regulatory governance does not suggest it to be important in explaining associations between population residential proximity to wells and county-level income, although countries with lower regulatory governance show a slightly positive trend. Future research could evaluate how laws, regulation and permitting processes are important in shaping the distribution of oil and gas wells across nations (56).

Citizen participation is also important in shaping the environmental licensing process for oil and gas (57). Some countries give local communities the right to decide whether oil and gas extraction occurs in their territory, while others are driven by financial interest and government corruption (2). A comparative study of unconventional gas development in the U.K. and Australia found that differing governance structures shape distributive and procedural justice outcomes (52). In the U.K., local governments hold decision-making power, while in Australia, authority lies with the state, limiting local influence. These power dynamics may explain why more exposed regions in Australia had lower incomes. Such differences illustrate how governance and community participation influence the unequal distribution of environmental burdens and benefits. This pattern has also been seen in Brazil’s Amazon, where oil and gas projects have excluded Indigenous populations (24). It was outside the scope of the current study to examine distributive justice issues among Indigenous communities, and further work could help determine whether there are such disparities across the study region.

Our study has several important limitations to consider when interpreting our results. First, the study is descriptive in nature and although patterns can generate hypotheses regarding how the oil and gas industry operates in each national context, we cannot definitively identify underlying mechanisms or quantify the public health burden of this industry. Second, our population counts likely underestimate the true population that resides in close proximity to oil and gas development. For example, the GHSL population data may not fully capture camps and work sites where oil and gas industry employees may temporarily reside, further contributing to underestimation. Third, we are unable to disentangle some characteristics of the oil and gas wells, such as active status or drilling type, a common limitation of large-scale datasets (32,58). This is particularly relevant because abandoned and orphaned wells can continue to release gas containing VOCs, and orphaned well leaks have been shown to exceed fugitive emissions from production equipment (59). Additionally, because we do not evaluate changes in well activity over time, excluding wells based on their current status could overlook prior exposure during periods when wells were active. Fourth, some oil and gas wells are located across country borders, introducing uncertainty in the attribution of national ownership and regulatory oversight. Fifth, aggregating exposure and social data at the ADM2 level may not adequately capture local disparities in some settings. Finally, residential proximity serves only as a proxy for population exposure, and differences in industrial emissions, wind patterns, housing characteristics, and population behavior may result in substantial variation in individual exposure.

Our findings provide an important foundation for future research examining population exposure to oil and gas development, particularly in understudied regions. Future studies should incorporate information on well characteristics, including activity status, drilling type, and temporal changes in operations, to better characterize exposure over time. Given that exposure pathways and potential mechanisms may differ between natural gas and oil wells, studies should also examine these well types separately and evaluate differences in the populations living near them. More spatially resolved data could help identify local disparities that may be obscured at the ADM2 level, while incorporating meteorological conditions, emissions data, housing characteristics, and population mobility could improve estimates of individual-level exposure. Finally, statistical modeling and longitudinal data could help evaluate associations between oil and gas development and health outcomes and provide more robust estimates of the potential public health burden. Together, these approaches could build on our descriptive findings to better understand how oil and gas development affects health across diverse national contexts.

In conclusion, our findings demonstrate that residential proximity to oil and gas wells is a widespread phenomenon that extends far beyond high income regions where most related health impacts have been studied to date. By quantifying the number of people living near extraction activities and identifying social disparities across 33 countries, this analysis highlights a substantial burden and inequities in who bears the environmental and health risks of global energy production. These findings may suggest that socioeconomic and political factors influence where oil and gas infrastructure is sited, contributing to unequal environmental burdens. We hope this provides an evidence base for future epidemiological research to estimate the magnitude of health impacts of this global industry. As demand for oil and gas persists and grows, particularly in low- and middle-income regions, these burdens are likely to intensify without coordinated action. Global efforts to monitor, regulate, and ultimately transition away from fossil fuel extraction must account for the communities most affected, ensuring that the benefits and harms of the energy system are distributed equitably.

## Supplementary Material

Supplementary materials

## Figures and Tables

**Figure 1:**
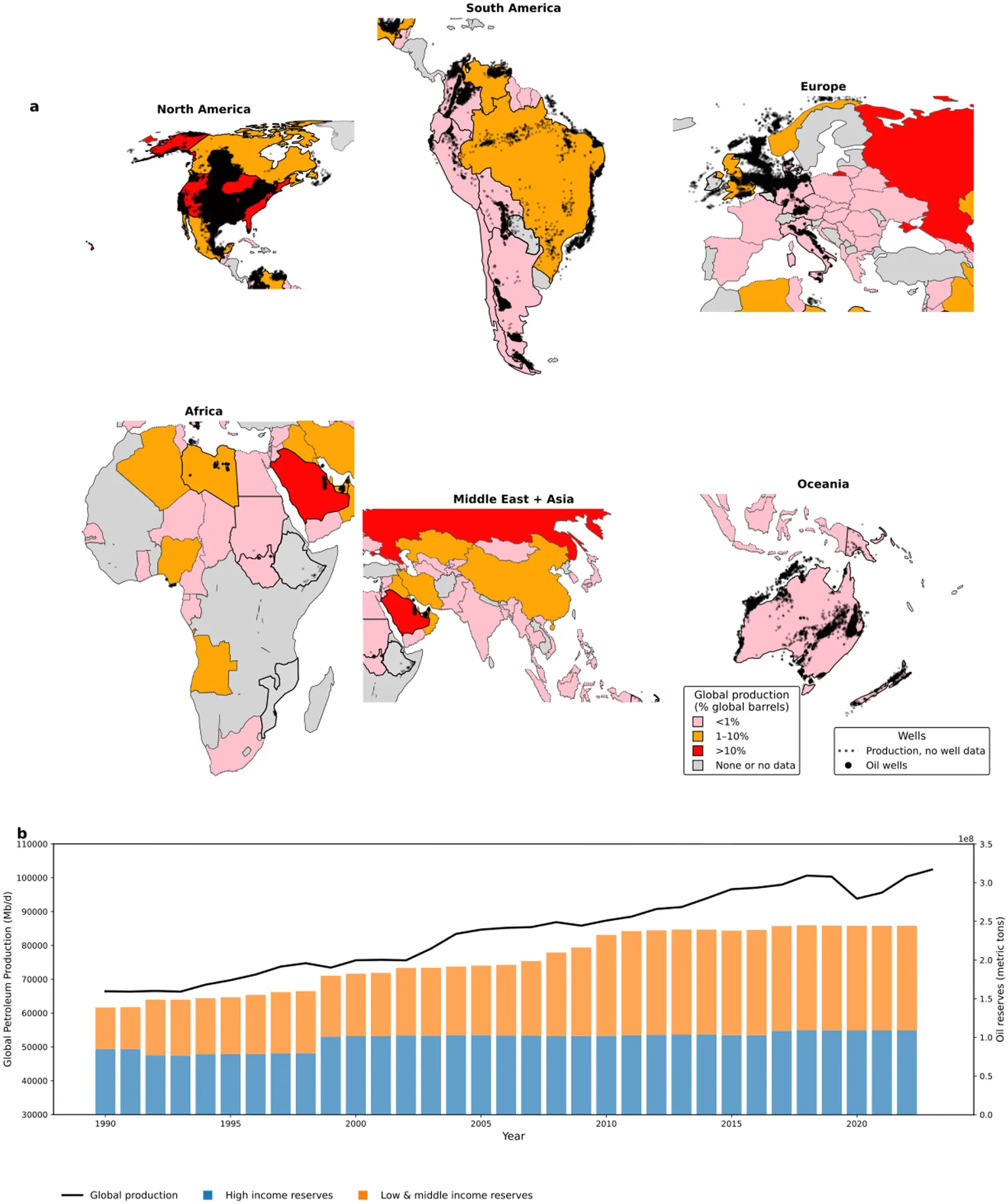
Oil and gas extraction by country and changes through time. a) map of oil and gas wells across 33 countries from the Oil and Gas Infrastructure Mapping Database (OGIM) with outline of crude oil production (% of global barrels per day) from the U.S. Energy Information Administration (EIA), December 2024; b) global oil petroleum production from EIA and total global crude oil reserves for high income and low- and middle- income countries from United Nations Statistics Division Energy Statistics Database (by WBG classification), 1985–2022.

**Figure 2:**
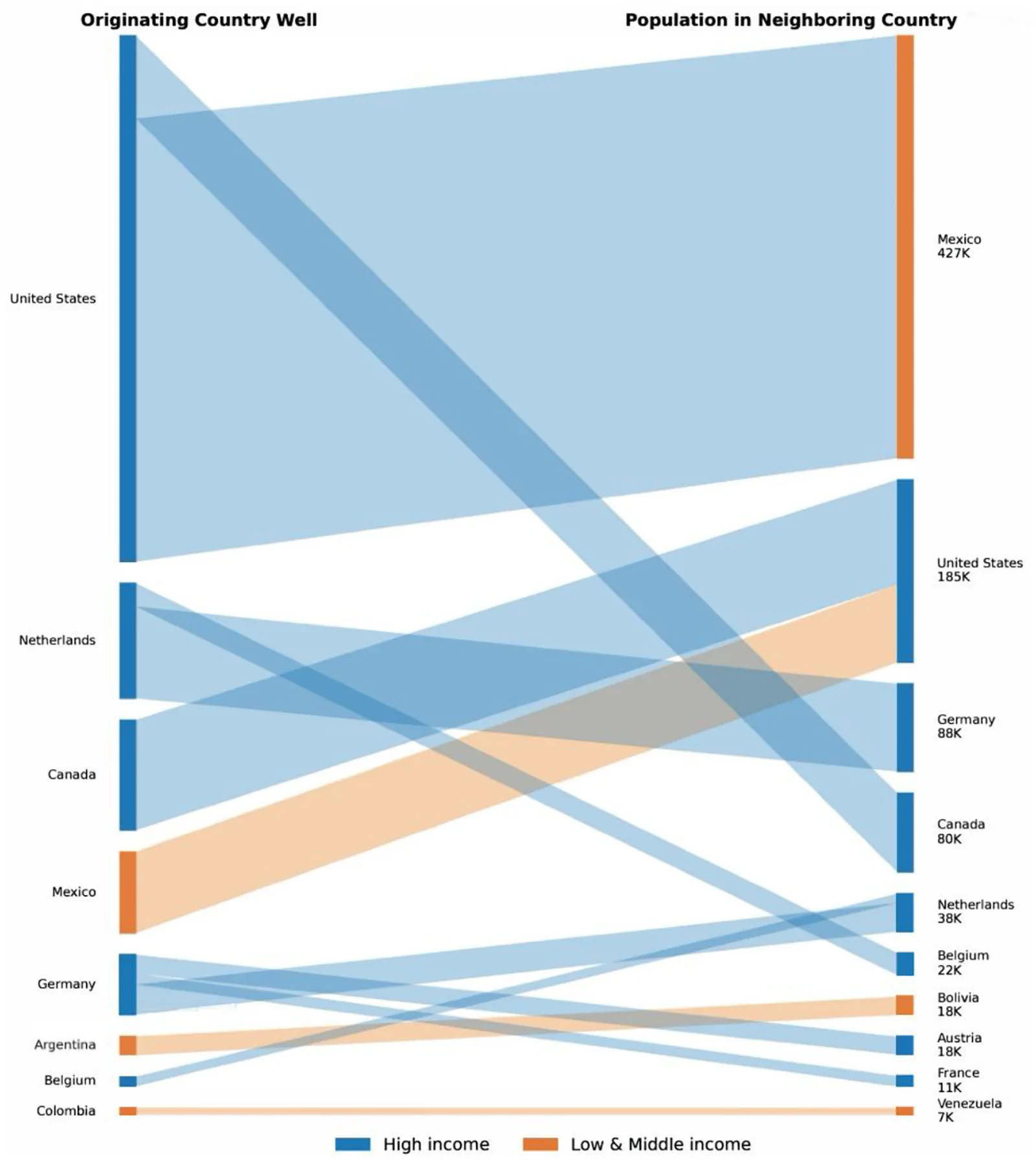
Population living within 3km of oil and gas wells from neighboring country colored by country income group (high income and low and middle income) for the 10 countries with highest residential proximity to out-of-country wells.

**Figure 3:**
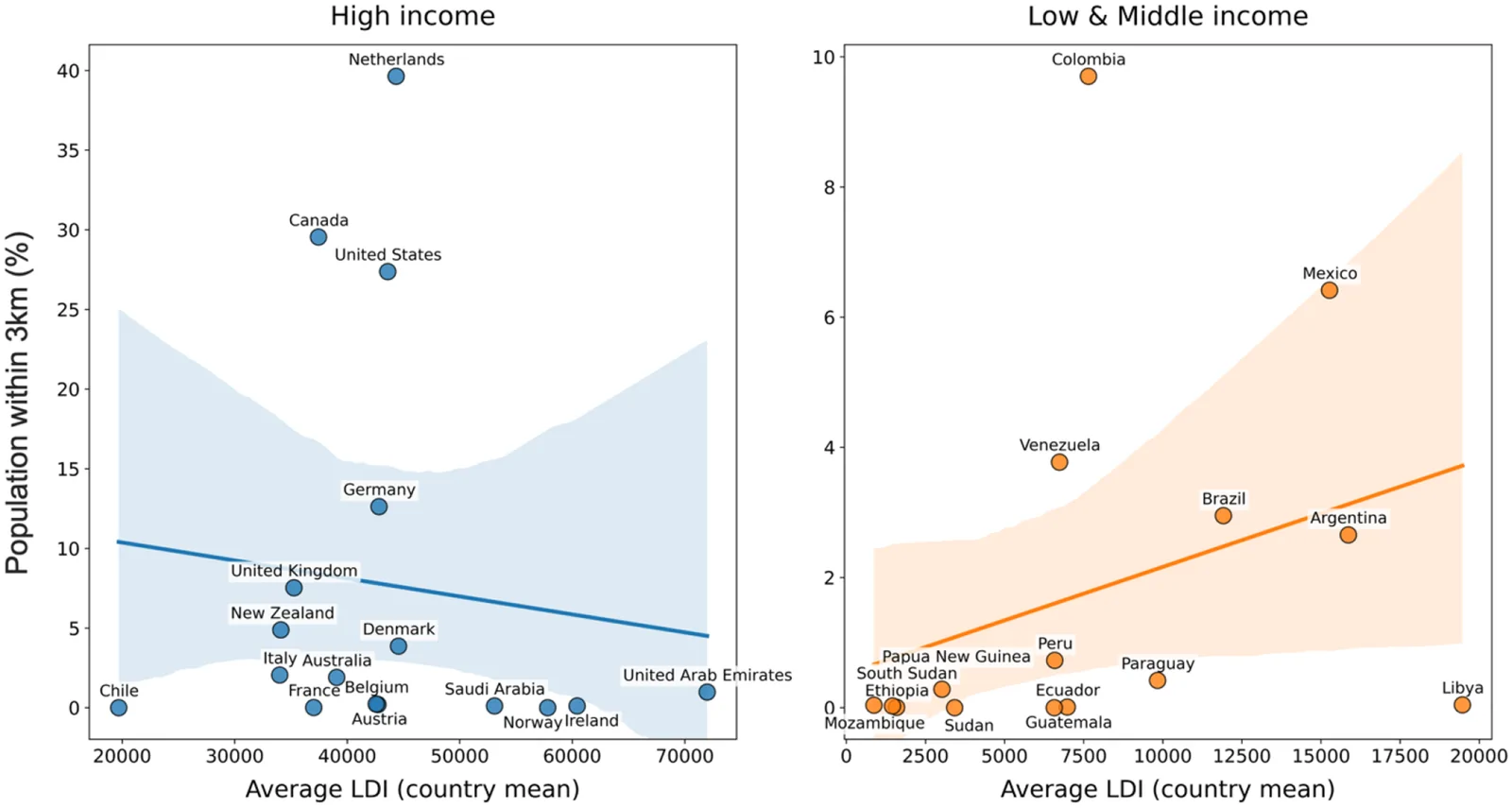
Country-level residential proximity to oil and gas wells (within 3km) and mean estimates of lag-distributed income (LDI) per capita (expressed in real 2010 purchasing power parity-adjusted dollars) by World Bank Income Group classification. *High income:* Australia, Austria, Belgium, Canada, Chile, Denmark, France, Germany, Ireland, Italy, Netherlands, New Zealand, Saudi Arabia, United Arab Emirates, United Kingdom, United States. *Low & Middle income:* Argentina, Bolivia, Brazil, Colombia, Ecuador, Ethiopia, Guatemala, Libya, Mexico, Mozambique, Papua New Guinea, Paraguay, Peru, South Sudan, Sudan.

**Figure 4:**
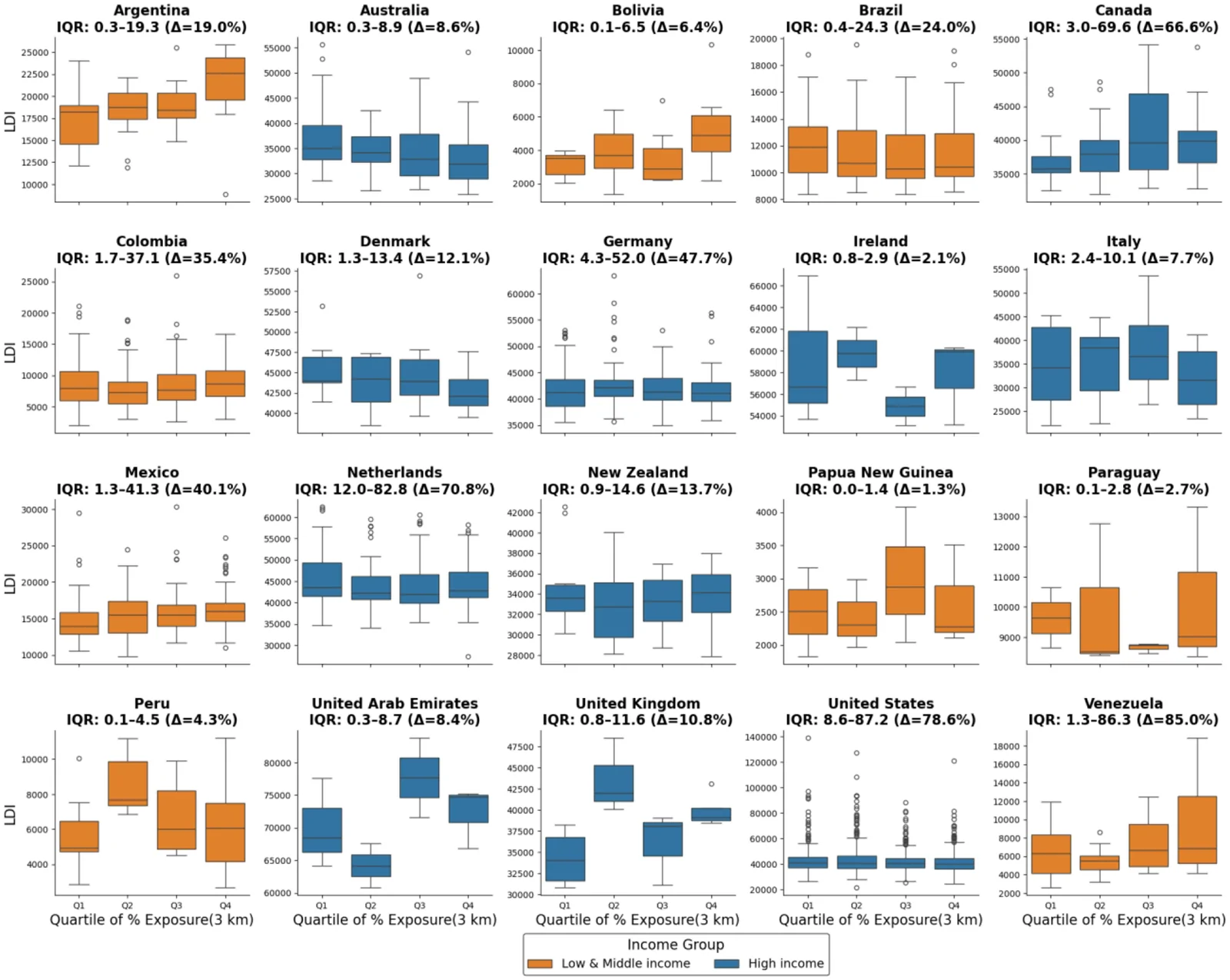
Association between lag-distributed income per capita (LDIpc) oil and within-country quartile of residential proximity to oil and gas (within 3km).^1^

**Figure 5:**
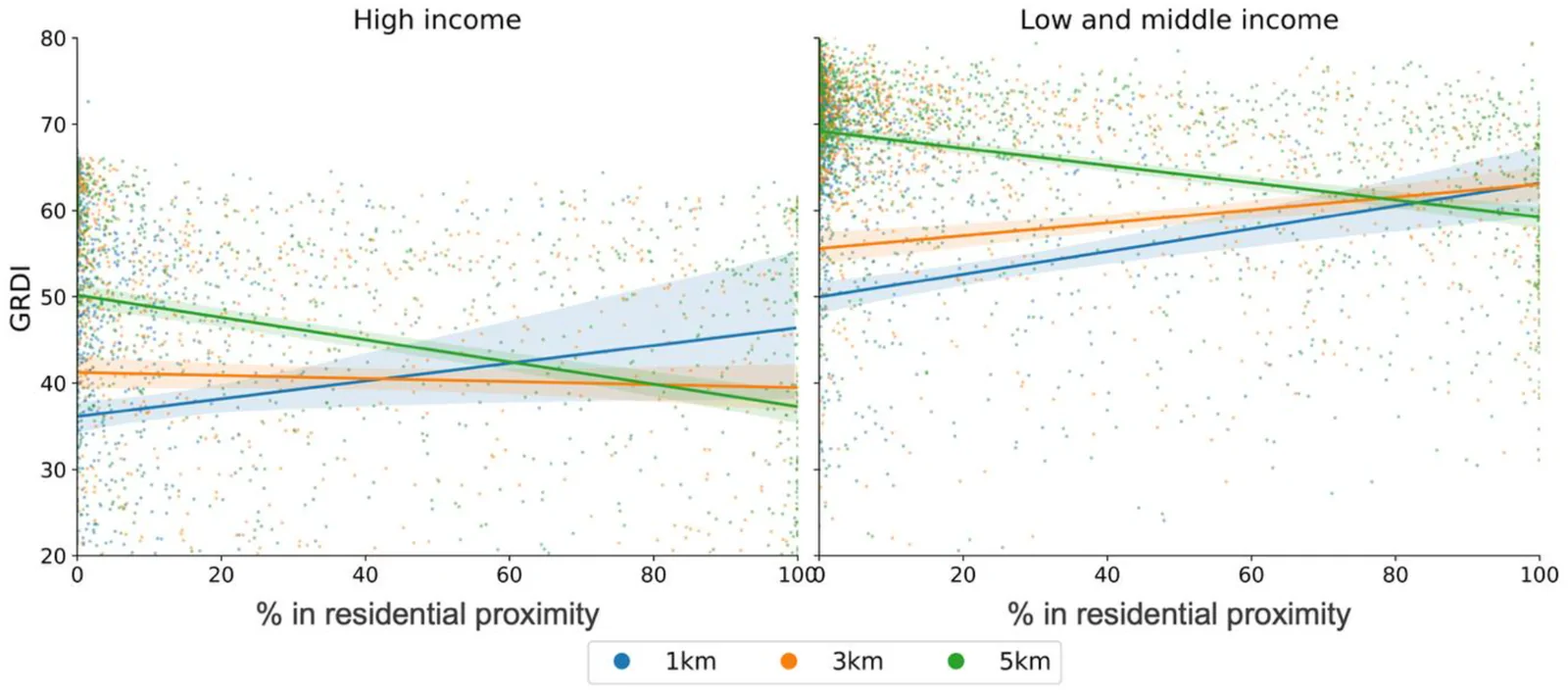
Association between average gridded relative deprivation index (GRDI) and population in residential proximity to oil and gas for different distances from well (1km, 3km, 5km) for high income and low and middle income countries. *High income:* Australia, Austria, Belgium, Canada, Chile, Denmark, France, Germany, Ireland, Italy, Netherlands, New Zealand, Saudi Arabia, United Arab Emirates, United Kingdom, United States. *Low & Middle income:* Argentina, Bolivia, Brazil, Colombia, Ecuador, Ethiopia, Libya, Mexico, Mozambique, Papua New Guinea, Paraguay, Peru, South Sudan, Sudan.

**Table 1:** Total population residing near (1km, 3km & 5km) an oil and gas well across 33 countries in order of 3km residential proximity.

Country	Population exposed N (%)		
	1km	3km	5km
**Netherlands**	1,701,479 (9.77)	6,905,557 (39.64)	10,739,553 (61.65)
**Canada**	3,783,123 (10.09)	11,080,372 (29.54)	15,783,155 (42.08)
**United States**	42,863,229 (12.76)	91,937,291 (27.37)	120,501,037 (35.88)
**Germany**	2,888,837 (3.46)	10,528,660 (12.63)	17,438,534 (20.91)
**Colombia**	968,416 (1.90)	4,939,288 (9.70)	10,018,876 (19.68)
**Bolivia**	261,944 (2.19)	1,120,762 (9.39)	1,892,651 (15.86)
**United Kingdom**	624,921 (0.93)	5,050,640 (7.54)	11,359,301 (16.95)
**Mexico**	2,725,899 (2.16)	8,074,221 (6.41)	13,320,788 (10.58)
**New Zealand**	43,174 (0.86)	246,449 (4.89)	479,708 (9.51)
**Denmark**	34,625 (0.60)	224,640 (3.87)	708,890 (12.20)
**Venezuela**	563,788 (1.98)	1,074,648 (3.77)	1,415,241 (4.97)
**Brazil**	2,129,701 (1.00)	6,287,276 (2.95)	9,191,201 (4.31)
**Argentina**	515,273 (1.14)	1,196,981 (2.66)	2,079,044 (4.61)
**Italy**	168,905 (0.28)	1,220,952 (2.05)	2,886,295 (4.85)
**Australia**	91,132 (0.36)	487,962 (1.90)	1,068,406 (4.17)
**United Arab Emirates**	12,601 (0.14)	90,946 (0.99)	192,601 (2.09)
**Peru**	142,000 (0.43)	240,536 (0.73)	443,554 (1.34)
**Paraguay**	6,399 (0.10)	27,777 (0.42)	34,599 (0.52)
**Papua New Guinea**	1,649 (0.02)	27,536 (0.28)	37,695 (0.39)
**Belgium**	691 (0.01)	26,568 (0.23)	96,851 (0.84)
**Austria**	17 (0.00)	18,234 (0.20)	42,435 (0.48)
**Ireland**	846 (0.02)	5,419 (0.11)	15,106 (0.31)
**Saudi Arabia**	12,499 (0.03)	40,378 (0.11)	51,563 (0.14)
**Libya**	1,599 (0.02)	2,959 (0.04)	3,112 (0.05)
**Mozambique**	1,880 (0.01)	12,069 (0.04)	18,691 (0.06)
**South Sudan**	1,400 (0.01)	2,896 (0.03)	5,035 (0.05)
**France**	789 (0.00)	10,532 (0.02)	14,064 (0.02)
**Ecuador**	801 (0.00)	1,695 (0.01)	2,449 (0.01)
**Ethiopia**	2,462 (0.00)	6,083 (0.01)	10,189 (0.01)
**Chile**	6 (0.00)	55 (0.00)	129 (0.00)
**Sudan**	0 (0.00)	115 (0.00)	466 (0.00)
**Norway**	0 (0.00)	67 (0.00)	80 (0.00)
**Guatemala**	0 (0.00)	0 (0.00)	664 (0.00)

## Data Availability

All data used in this study is publicly available. Further information about the datasets and codes used is available at the following link: https://github.com/nina-flores/ogd.

## Footnotes

1 Austria, Belgium, Chile, Ecuador, Ethiopia, France, Guatemala, Libya, Norway, Mozambique, Saudi Arabia, South Sudan, Sudan excluded due to insufficient variability (<6 ADM2-level units).
