# Supplementary material for: Residential proximity to oil and gas development and socioeconomic conditions: A comparative analysis of 33 countries
Source: Environ Res Lett. Author manuscript; Available in PMC 2026 Nov 11. (PMC13750840; doi:10.1088/1748-9326/aeae53)
Supplement: Supplementary file 1 [file NIHMS2217418-supplement-Supplementary_materials.docx]

**Residential proximity to oil and gas development and socioeconomic conditions: A comparative analysis of 33 countries**

Lara Schwarz,^1^ Nina M. Flores,^2^ David J.X. González,^1^ Heather McBrien,^2^ Nina Brooks,^3^ Jonathan J. Buonocore,^4^ Amira Aker,^5^ Veronica Southerland,^6^ Joan A. Casey,^7,8^ Mary D Willis^5^

1 Department of Environmental Health Sciences, University of California Berkeley School of Public Health, Berkeley, CA, USA

2 Department of Environmental Health Sciences, Mailman School of Public Health, Columbia University, New York, NY, USA

3 School for Environment and Sustainability, University of Michigan, Ann Arbor, MI, USA

4 Department of Environmental Health, Boston University School of Public Health, Boston, MA, USA

5 Department of Epidemiology, Boston University School of Public Health, Boston, MA, USA

6 Environmental Defense Fund, Washington D.C., USA

7 Department of Environmental Health and Occupational Health Sciences, School of Public Health, University of Washington, Seattle, WA, USA

8 Department of Epidemiology, School of Public Health, University of Washington, Seattle, WA, USA

**Table S1:** Data sources used in the study

| **Source** | **Description** |
| --- | --- |
| Oil and Gas Infrastructure Mapping database (OGIM) | Spatial features for important components of oil and gas infrastructure, including oil and gas extraction sites, using publicly available country-specific records of the oil and gas industry from 2024. |
| Global Human Settlement Layer | Gridded estimates at the 100m resolution of the spatial distribution of residential population estimated using spatial-temporal interpolation of observed collections of multiple-sensor, multiple-platform satellite imageries including Landsat satellite data for 2020. |
| IHME Lag-distributed  Income and Socio-Demographic Index | Administrative II level estimates of relative poverty using relevant data on household wealth from various sources. This is a measure of wealth defined as a moving average transformation of per person income with a 10-year lag and is expressed in 2010 purchasing power parity-adjusted dollars from 2024. |
| MOD44W.006 Terra Land Water Mask Derived from MODIS and SRTM Yearly Global | This land/water mask 250m product is derived using a decision tree classifier trained with MODIS data and validated with the MOD44W V5 product. It identifies areas that are water at a global scale. Estimates for the year 2015 were used. |
| World Bank Country Income Classifications | All countries in the world are classified using standardized methods into the following categories: low and middle (low, lower-middle, upper-middle), and high. |
| Global Gridded Relative Deprivation Index (GRDI) V1 | Relative levels of multidimensional deprivation and poverty in each 30 arc-second (~1 km) pixel, where a value of 100 represents the highest level of deprivation and a value of 0 the lowest. Input dimensions range from 2010 to 2020. |

**Table S2**: Number of total wells, offshore wells and out of country wells (located in a bordering country but within 5km of country border) by country.

| **Country** | **Wells (n)** | | |
| --- | --- | --- | --- |
|  | **Total** | **Offshore** | **Out of country** |
| **Argentina** | 83,629 | 378 | 55 |
| **Australia** | 26,070 | 325 | - |
| **Austria** | 36 | - | 36 |
| **Belgium** | 47 | - | 44 |
| **Bolivia** | 2,067 | 3 | 71 |
| **Brazil** | 24,139 | 985 | - |
| **Canada** | 631,404 | 3,962 | 3,754 |
| **Chile** | 379 | 10 | 365 |
| **Colombia** | 24,924 | 92 | 40 |
| **Denmark** | 209 | 6 | 2 |
| **Ecuador** | 77 | - | 72 |
| **Ethiopia** | 19 | - | - |
| **France** | 54 | - | 54 |
| **Guatemala** | 1 | - | 1 |
| **Germany** | 21,863 | 179 | 1,232 |
| **Ireland** | 13 | - | - |
| **Italy** | 1,387 | 55 | - |
| **Libya** | 384 | - | - |
| **Mexico** | 34,991 | 287 | 3,370 |
| **Mozambique** | 32 | - | - |
| **Netherlands** | 5,492 | 110 | 1,048 |
| **New Zealand** | 1,015 | 22 | - |
| **Norway** | 1 | 1 | - |
| **Papua New Guinea** | 35 | - | - |
| **Paraguay** | 54 | - | 2 |
| **Peru** | 5,992 | 192 | 1 |
| **Saudi Arabia** | 2,005 | - | 1 |
| **South Sudan** | 40 | - | 1 |
| **Sudan** | 3 | - | - |
| **United Arab Emirates** | 691 | - | - |
| **United Kingdom** | 2,144 | 79 | 5 |
| **United States** | 3,596,365 | 38,367 | 3,906 |
| **Venezuela** | 20,613 | 453 | 959 |

**Table S3:** Population exposure to offshore and out of country wells by country.

| **Country** | Exposed Population, 3km (n) | | | |
| --- | --- | --- | --- | --- |
|  | **Offshore** | **Out-of-country total** | **Out-of-country 1** | **Out-of-country 2** |
| **Argentina** | 165,072 | 3,661 | Bolivia (3,617) | Chile (44) |
| **Australia** | 2,497 | - | - | - |
| **Austria** | - | 18,234 | Germany (18,234) | - |
| **Belgium** | - | 22,351 | Netherlands (22,351) | - |
| **Bolivia** | 80 | 18,414 | Argentina (18,414) | - |
| **Brazil** | 1,090,354 | - | - | - |
| **Canada** | 271,721 | 79,973 | United States (79,973) | - |
| **Chile** | - | 31 | Argentina (31) | - |
| **Colombia** | 145,183 | 2,659 | Venezuela (2,238) | Ecuador (421) |
| **Denmark** | 37,378 | - | - | - |
| **Ecuador** | - | 1,448 | Colombia (1,448) | - |
| **Ethiopia** | - | - | - | - |
| **France** | - | 10,532 | Germany (10,532) | - |
| **Germany** | 60,478 | 88,435 | Netherlands (88,435) | - |
| **Guatemala** | - | - | - | - |
| **Ireland** | - | - | - | - |
| **Italy** | 2,555 | - | - | - |
| **Libya** | - | - | - | - |
| **Mexico** | 261,922 | 427,033 | United States (427,033) | - |
| **Mozambique** | - | - | - | - |
| **Netherlands** | 263,423 | 38,135 | Germany (29,061) | Belgium (9,074) |
| **New Zealand** | 36,763 | - | - | - |
| **Norway** | 67 | - | - | - |
| **Papua New Guinea** | - | - | - | - |
| **Paraguay** | - | - | - | - |
| **Peru** | 21,660 | - | - | - |
| **Saudi Arabia** | - | - | - | - |
| **South Sudan** | - | - | - | - |
| **Sudan** | - | - | - | - |
| **United Arab Emirates** | - | - | - | - |
| **United Kingdom** | 82,110 | 653 | Ireland (653) | - |
| **United States** | 5,035,875 | 184,598 | Canada (106,217) | Mexico (78,381) |
| **Venezuela** | 125,840 | 6,821 | Colombia (6,821) |  |

**Figure S1:** Subregional level units (ADM2) that have oil and gas exposure across 33 countries.

**Figure S2:** Country-level population exposure to oil and gas wells and mean estimates of lag-distributed income per capita (expressed in real 2010 purchasing power parity-adjusted dollars) by World Bank Income Group classification for each distance to oil and gas wells (1km, 3km, 5km).

**Figure S3:** Association between lag-distributed income per capita (LDI) oil and gas exposure (within 1km) by within-country quartile of population exposure for each country.

**Figure S4:** Association between lag-distributed income per capita (LDI) oil and gas exposure (within 5km) by within-country quartile of population exposure for each country.

**Figure S5:** Boxplot lag-distributed income (LDI) by quartile of exposed (3km exposure) with countries separated by WBG classification. *High income:* Australia, Austria, Belgium, Canada, Chile, Denmark, France, Germany, Ireland, Italy, Netherlands, New Zealand, Norway, Saudi Arabia, United Arab Emirates, United Kingdom, United States. *Low & Middle income:* Argentina, Bolivia, Brazil, Colombia, Ecuador, Ethiopia, Libya, Mexico, Mozambique, Papua New Guinea, Paraguay, Peru, South Sudan, Sudan, Venezuela. ^[1]^

**Figure S6:** Association between average gridded relative deprivation index (GRDI) and oil and gas exposure for different distances from well (1km, 3km, 5km) by country. ^[2]^

**Figure S7:** Country-level population residential proximity to oil and gas wells (within 3km) and mean estimates of lag-distributed income (LDI) per capita (expressed in real 2010 purchasing power parity-adjusted dollars) by a World Bank Global Indicator of Regulatory Governance (GIRG), split by higher regulatory governance (regulatory governance score (GIRG) ≥3.5) and lower regulatory governance (GIRG <3.5). Lower Regulatory Governance: Argentina, Bolivia, Chile, Ecuador, Ethiopia, Guatemala, Libya, Mozambique, Papua New Guinea, Paraguay, Peru, Saudi Arabia, South Sudan, Sudan, United Arab Emirates. Higher Regulatory Governance: Australia, Austria, Belgium, Brazil, Canada, Colombia, Denmark, France, Germany, Ireland, Italy, Mexico, Netherlands, New Zealand, Norway, United Kingdom, United States.^[3]^

1. Guatemala dropped because no population living within 3km of a well.
2. Austria, Belgium, Chile, Ecuador, Ethiopia, France, Guatemala, Libya, Mozambique, Norway, Saudi Arabia, South Sudan, Sudan excluded due to insufficient within-country variability (<6 ADM2-level units).
3. Venezuela excluded due to missing from World Bank Global Indicator of Regulatory Governance data.
